# PEEP Titration Is Markedly Affected by Trunk Inclination in Mechanically Ventilated Patients with COVID-19 ARDS: A Physiologic, Cross-Over Study

**DOI:** 10.3390/jcm12123914

**Published:** 2023-06-08

**Authors:** Francesco Marrazzo, Stefano Spina, Francesco Zadek, Clarissa Forlini, Gabriele Bassi, Riccardo Giudici, Giacomo Bellani, Roberto Fumagalli, Thomas Langer

**Affiliations:** 1 Department of Anesthesia and Critical Care, ASST Grande Ospedale Metropolitano Niguarda, 20162 Milano, Italy; francesco.marrazzo@ospedaleniguarda.it (F.M.); stefano.spina@ospedaleniguarda.it (S.S.); clarissa.forlini@ospedaleniguarda.it (C.F.); gabriele.bassi@ospedaleniguarda.it (G.B.); riccardo.giudici@ospedaleniguarda.it (R.G.); roberto.fumagalli@unimib.it (R.F.); 2 School of Medicine and Surgery, University of Milano-Bicocca, 20126 Milano, Italy; francesco.zadek@unimib.it (F.Z.); giacomo.bellani@apss.tn.it (G.B.); 3 Department of Anesthesia and Intensive Care 1, Santa Chiara Hospital, APSS Trento, 38122 Trento, Italy

**Keywords:** acute respiratory distress syndrome, mechanical ventilation, trunk inclination, COVID-19, ventilator-induced lung injury

## Abstract

Background: Changing trunk inclination affects lung function in patients with ARDS. However, its impacts on PEEP titration remain unknown. The primary aim of this study was to assess, in mechanically ventilated patients with COVID-19 ARDS, the effects of trunk inclination on PEEP titration. The secondary aim was to compare respiratory mechanics and gas exchange in the semi-recumbent (40° head-of-the-bed) and supine-flat (0°) positions following PEEP titration. Methods: Twelve patients were positioned both at 40° and 0° trunk inclination (randomized order). The PEEP associated with the best compromise between overdistension and collapse guided by Electrical Impedance Tomography (PEEP_EIT_) was set. After 30 min of controlled mechanical ventilation, data regarding respiratory mechanics, gas exchange, and EIT parameters were collected. The same procedure was repeated for the other trunk inclination. Results: PEEP_EIT_ was lower in the semi-recumbent than in the supine-flat position (8 ± 2 vs. 13 ± 2 cmH_2_O, *p* < 0.001). A semi-recumbent position with optimized PEEP resulted in higher PaO_2_:FiO_2_ (141 ± 46 vs. 196 ± 99, *p* = 0.02) and a lower global inhomogeneity index (46 ± 10 vs. 53 ± 11, *p* = 0.008). After 30 min of observation, a loss of aeration (measured by EIT) was observed only in the supine-flat position (−153 ± 162 vs. 27 ± 203 mL, *p* = 0.007). Conclusions: A semi-recumbent position is associated with lower PEEP_EIT_ and results in better oxygenation, less derecruitment, and more homogenous ventilation compared to the supine-flat position.

## 1. Introduction

Invasive mechanical ventilation is a lifesaving support in patients with acute respiratory distress syndrome (ARDS). Its implementation is challenging since it can further deteriorate lung function (ventilator-induced lung injury—VILI) [1]. To reduce the incidence of VILI, several measures can be adopted [2,3]. On the one hand, these measures can operate on the ventilator through individualization of ventilator settings, such as tidal volume and positive end-expiratory pressure (PEEP) [4,5]. On the other, they operate to improve the patient-ventilator interaction by changing patients’ postures, such as during prone position [6,7]. A less investigated postural factor is the angle of trunk inclination in supine, mechanically ventilated patients [8,9,10]. Indeed, in this context, variations in trunk inclination have a marked effect on respiratory mechanics [8,9,11,12]. We recently described an increase in respiratory system compliance in patients with COVID-19-associated ARDS (C-ARDS) when trunk inclination was changed from a semi-recumbent to a supine-flat position at a constant ventilatory setting. In addition, the supine-flat position improved carbon dioxide (CO_2_) clearance, suggesting the occurrence of alveolar hyperinflation in the semi-recumbent position [11]. Given these premises, one could hypothesize that the incidence of VILI might be reduced with the use of a supine-flat position. However, a trunk inclination up to 45° is suggested by the bundles for ventilator-associated pneumonia prevention [13].

As we hypothesized that the “best” PEEP [14] might be markedly affected by trunk inclination, we investigated whether similar positive effects on lung mechanics and gas exchange, observed in the supine-flat position, could be obtained in a semi-recumbent position with optimized PEEP.

The primary aim of the present study was therefore to evaluate whether there is a difference between “best PEEP” titrated with Electrical Impedance Tomography (EIT) in supine-flat and semi-recumbent positions [14,15]. The secondary aim was to evaluate the impact of trunk inclination on respiratory mechanics, gas exchange, and EIT parameters following PEEP optimization.

## 2. Materials and Methods

### 2.1. Study Population

This is a single-center, cross-over, physiologic study. Patients were consecutively enrolled from February 2022 to March 2022. The study was approved by the institutional review board (Ref# 82-16022022) and informed consent was obtained according to Italian regulations. The inclusion and exclusion criteria were the following:

### 2.2. Inclusion Criteria

- Laboratory-confirmed SARS-CoV-2 infection
- Diagnosis of ARDS according to Berlin definition [16]
- Deep sedation, paralysis, and controlled mechanical ventilation

### 2.3. Exclusion Criteria

- Age < 18 years
- Contraindications to recruitment maneuver due to one of the following conditions: hemodynamic instability (defined as mean artery pressure < 60 mmHg with norepinephrine > 0.3 mg/kg/min), emphysema with subpleural pulmonary bullae, pneumomediastinum, bronchopleural fistulae
- Contraindications to the placement of EIT belts (i.e., implantable cardiac pacemaker or defibrillator, trauma or burn of the chest wall) [17,18]
- Contraindications to mobilization (i.e., unstable spinal injury, intracranial hypertension)
- Pregnancy

At study enrollment, demographic and clinical data were collected.

### 2.4. Respiratory Monitoring

Patients were on volume-controlled ventilation (Dräger Evita V800, Dräger Medical, Lübeck, Germany). A 5-French esophageal balloon (CooperSurgical, Trumbull, CT, USA) was placed, and its correct position checked by Baydur maneuver [19]. A 16-electrode EIT belt was placed around the chest (between the 4th and 5th intercostal space) and connected to a dedicated monitor [20]. Airway pressure, esophageal pressure, and EIT images were continuously recorded and stored for subsequent offline analyses (Pulmovista 500 and Pressure Pod, Dräger Medical, Lübeck, Germany).

### 2.5. Study Protocol

All patients underwent two steps where trunk inclination was changed from semi-recumbent to supine-flat or vice versa (cross-over design), depending on randomization.

Opaque sealed envelopes and a block randomization were used to ensure the balance of the starting position (half of the envelopes consisting of a first step in the supine-flat position and a second step in the semi-recumbent position, and the other half consisting of a first step in the semi-recumbent position and a second step in the supine-flat position). Once patients were considered eligible, the opaque envelope was randomly drawn immediately prior to starting any procedure. Based on the recent literature [11], we did not expect a carry-over effect of the starting position. However, the randomization guaranteed a balanced starting position, eliminating this potential confounding factor.

**Figure 1:**
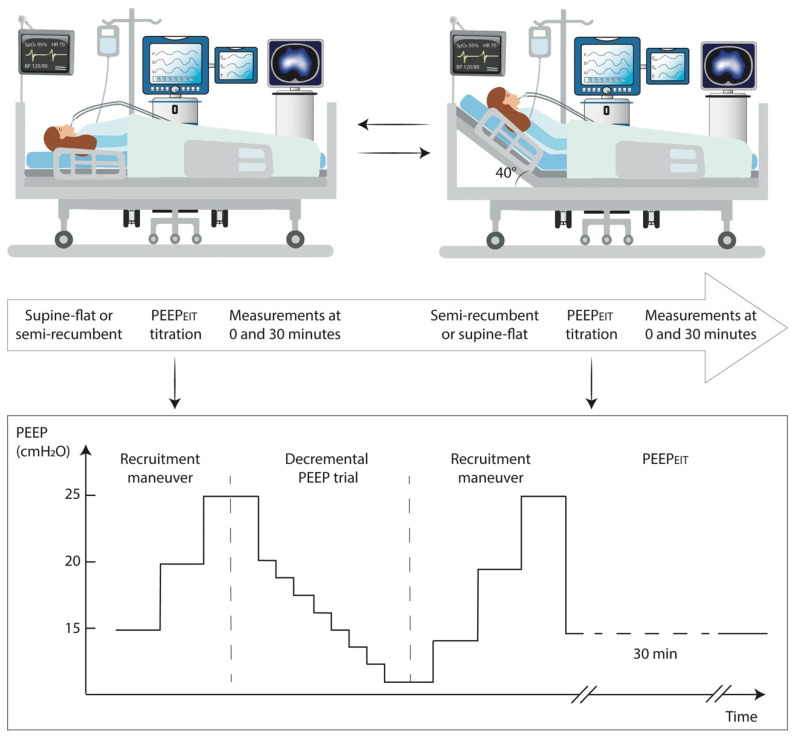
Study protocol.

At the beginning of each step, the proper function of the esophageal balloon was reassessed. Then, a recruitment maneuver was performed with pressure-controlled ventilation with a respiratory rate of 10 breaths/min, an inspiratory to expiratory ratio of 1:1, and a pressure control of 15 cmH_2_O. Initially, PEEP was set at 15 cmH_2_O and then increased by 5 cmH_2_O every 30 s, up to a PEEP of 25 cmH_2_O. A decremental PEEP trial was then performed with volume-controlled ventilation, maintaining the tidal volume set by clinicians. PEEP was initially set at 20 cmH_2_O and reduced stepwise by 2 cmH_2_O down to a minimum PEEP value of 6 cmH_2_O. The recruitment maneuver was promptly interrupted if severe hypotension developed (i.e., systolic blood pressure < 70 mmHg or mean artery pressure < 40 mmHg) or if peripheral oxygen saturation (SpO_2_) decreased below 88% despite a progressive increase in FiO_2_ up to 1.0. At each PEEP level, the percentage of lung overdistension and collapse were calculated by comparing pixel compliances at different PEEP levels and then displayed with EIT. The PEEP value associated with the best compromise of overdistension and collapse was defined as the best PEEP by using EIT (PEEP_EIT_, primary endpoint), as previously described [14,15,21]. A constant inspiratory flow (40 L/minute) was set, and a brief constant inspiratory hold (0.4 s) was obtained. Thus, driving pressure during the decremental PEEP trial was also calculated. After an additional recruitment maneuver, PEEP_EIT_ was set and ventilation continued for 30 min without changing trunk inclination. Partitioned respiratory mechanics, blood gas analysis, and basic hemodynamics were recorded thereafter (secondary endpoints). Then, trunk inclination was changed and the entire procedure was repeated (Figure 1).

All 12 patients underwent 2 30 min steps. Ventilatory settings were kept constant throughout the study in all patients, except for PEEP, which was set according to the EIT-based PEEP titration. At the end of each study step, we measured/calculated:

- Vital signs: heart rate; blood pressure; central venous pressure; peripheral oxygen saturation.
- Partitioned respiratory mechanics: tidal volume; respiratory rate; mean airway pressure; total positive end-expiratory pressure; plateau pressure; driving pressure; esophageal pressure at end-inspiration and at end-expiration; transpulmonary pressure at end-inspiration and at end-expiration; driving transpulmonary pressure; respiratory system, chest wall, and lung compliance.
- Venous and arterial blood gas analysis: pH; SaO_2_; SvO_2_; PaO_2_:FiO_2_; oxygenation index; PaCO_2_; ∆PvCO_2_-PaCO_2_; venous admixture; alveolar dead space; ventilatory ratio; lactates.
- Electrical Impedance Tomography: EIT data were acquired and stored throughout the study; end-expiratory lung impedance (EELI); change in end-expiratory lung volume (EELV) based on EELI (see below); percentage of ventilation distribution in the ventral and dorsal regions; regional respiratory system compliance (ventral and dorsal); global inhomogeneity index [22,23].

EIT images were continuously recorded and divided into two horizontal regions of interest of similar size to compute ventilation distribution and regional compliance of the dorsal and ventral lung areas [24]. The Global inhomogeneity index was recorded [22]. Variations in end-expiratory lung volume (EELV) were calculated from changes in end-expiratory lung impedance (EELI) recorded during the 30-min observation [25,26].

### 2.6. Sample Size Calculation

We calculated that a sample size of 12 would provide a power of 0.95 with an alfa-error of 0.05 to detect a difference in PEEP_EIT_ between the semi-recumbent and supine-flat positions of 3 cmH_2_O, assuming a standard deviation of 2.5 cmH_2_O, using a two-tailed paired t-student test. The expected mean difference of 3 ± 2 cmH_2_O was based on the difference in plateau pressure observed in a similar setting [11].

### 2.7. Statistical Analyses

Continuous variables are expressed as mean ± SD or median [IQR] as appropriate, while categorical variables are expressed as count and percentage. The normality of the distribution was tested using the Shapiro–Wilk test. Continuous variables were compared with the paired Student’s *t*-test or Mann–Whitney as appropriate. Statistical significance was defined as *p* < 0.05. Analysis was performed with Stata statistical software (StataCorp, College Station, TX, USA) and SigmaPlot (Systat Software, San Jose, CA, USA).

## 3. Results

Twelve patients were enrolled and studied 3 ± 2 days after intubation. Study subjects were 65 ± 8 years old, mainly non-obese, predominantly male and on protective mechanical ventilation settings (Table 1).

**Figure 2:**
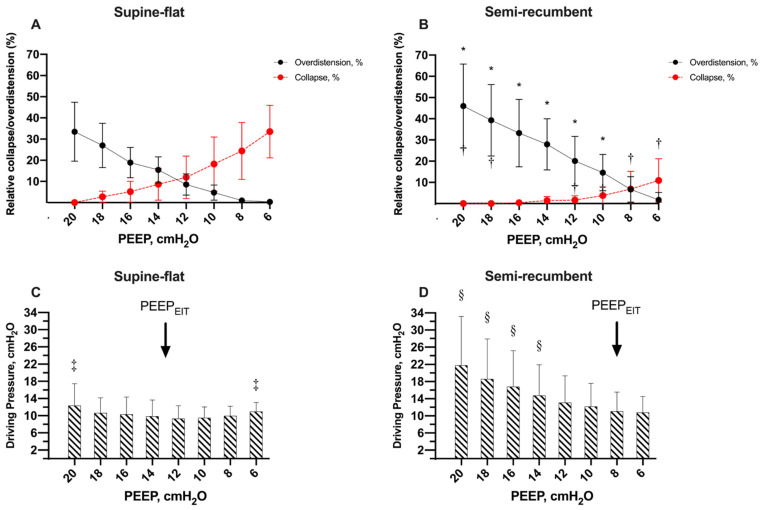
Overdistension, collapse and driving pressure during PEEP titration performed in supine-flat and semi-recumbent positions. The results of collapse and overdistension during PEEP titration performed in supine-flat position and semi-recumbent position are presented in Panel (**A**) and Panel (**B**), respectively. Red dots represent the percentage of collapse, while black dots represent the percentage of overdistension. A two-way ANOVA for repeated measures was performed to evaluate the effect of trunk inclination and PEEP both on collapse and overdistension (*p* < 0.001 for both). * = *p* < 0.05 compared to the percentage of overdistension in supine-flat position for the same PEEP value; † = *p* < 0.05 compared to the percentage of collapse in supine-flat position for the same PEEP value. The values of driving pressure recorded during PEEP titration performed in supine-flat position and semi-recumbent position are presented in Panel (**C**) and Panel (**D**), respectively. A two-way ANOVA for repeated measures was performed to evaluate the effect of trunk inclination and PEEP on driving pressure (*p* < 0.001). ‡ = *p* < 0.05 compared to the best PEEP in supine-flat position (13 cmH_2_O). § = *p* < 0.05 compared to the best PEEP in semi-recumbent position (8 cmH_2_O).

**Figure 3:**
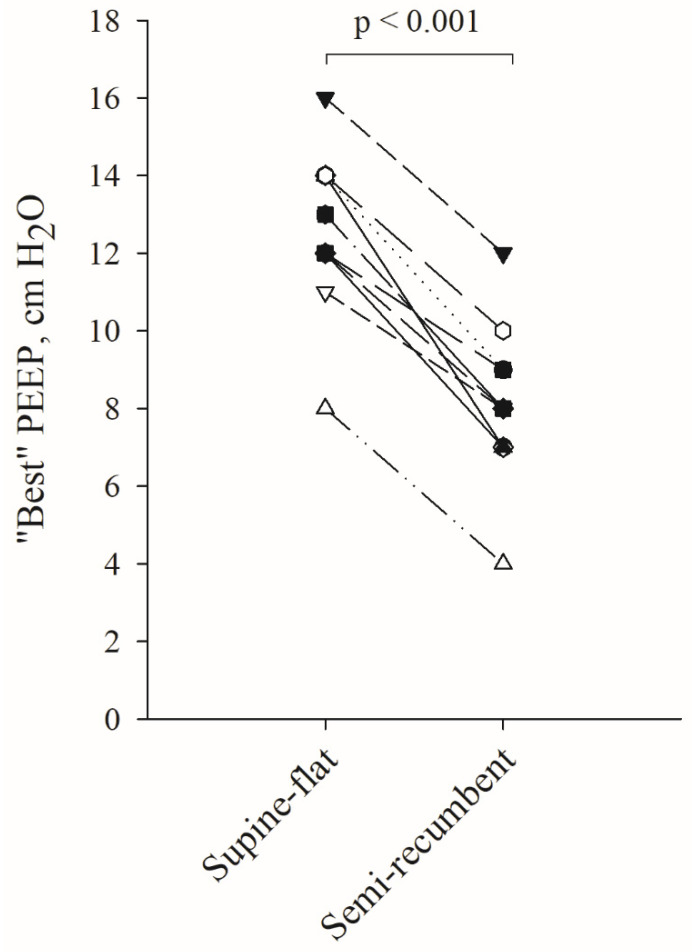
“Best” PEEP based on EIT in supine-flat and semi-recumbent position. Individual pairs of “best” PEEP identified through EIT in the two positions are reported. The *p*-value refers to the paired *t*-test. Data of 12 patients are reported; however, 2 pairs of patients have the same pair of “best” PEEP values in supine-flat and in semi-recumbent position. As a consequence, data of only 10 patients are visible.

The percentage of alveolar overdistension and collapse observed with EIT during the decremental PEEP trial differed markedly according to the patients’ trunk inclination (Figure 2A,B). Interestingly, the percentage of alveolar overdistension and collapse at PEEP_EIT_ were significantly higher in the supine-flat position (10 ± 5 vs. 5 ± 3%, *p* = 0.002 and 9 ± 5 vs. 5 ± 3%, *p* = 0.001, respectively). Moreover, alveolar collapse started at a higher PEEP value in the supine-flat position (at PEEP values between 18 and 16 cmH_2_O) compared to the semi-recumbent position (at PEEP values between 12 and 10 cmH_2_O) during the decremental PEEP trial. Conversely, alveolar overdistension appeared at very low PEEP values in the semi-recumbent position. As a result, PEEP_EIT_, i.e., the PEEP associated with the best compromise between alveolar distension and collapse, was significantly higher in the supine-flat position compared to the semi-recumbent position (13 ± 2 vs. 8 ± 2 cmH_2_O, *p* < 0.001; Figure 3). Furthermore, driving pressure calculated during the decremental PEEP trial was the lowest at the best PEEP_EIT_ (Figure 2C,D). This suggests a concordance between the best PEEP identified by EIT and the best PEEP associated with the highest respiratory system compliance.

**Figure 4:**
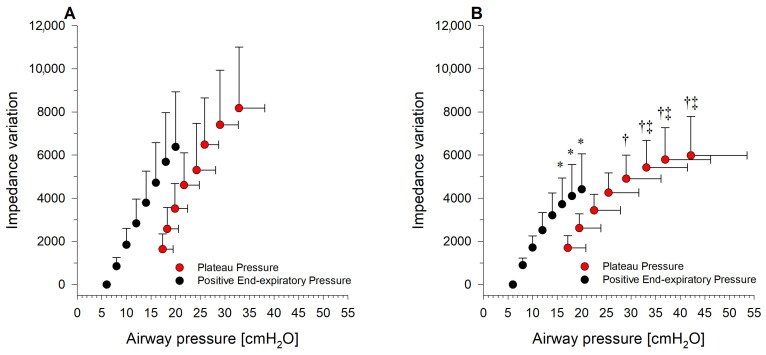
Pressure-Impedance variation curves in supine-flat and semi-recumbent position. Panel (**A**) Pressure-impedance variation curves recorded during the decremental PEEP trial in supine-flat position. Panel (**B**) Pressure-impedance variation curves recorded during the decremental PEEP trial in semi-recumbent position. In both panels, impedance represents changes in impedance compared to the impedance value measured at PEEP of 6 cmH_2_O. Data are presented as mean ± standard deviation. Notably, for data points referring to inspiratory pressure, the standard deviation is reported for both the change in lung impedance and plateau pressure. A two-way ANOVA for repeated measures was performed to evaluate the effect of trunk inclination (factor 1) and PEEP (factor 2) on end-inspiratory impedance variation (red dots, vertical standard deviation bars) and end-expiratory impedance variation (black dots) and plateau pressure (red dots, horizontal standard deviation bars) (*p* < 0.001 for all). * = *p* < 0.05 compared to end-expiratory impedance variation in supine-flat position for the same PEEP value; † = *p* < 0.05 compared to plateau pressure in supine-flat position for the same PEEP value; ‡ = *p* < 0.05 compared to end-inspiratory impedance variation in supine-flat position for the same PEEP value.

Lung impedance variations at end-inspiration and at end-expiration recorded during the decremental PEEP trial in the two positions are represented in Figure 4, Panel A and B). Notably, the semi-recumbent position showed a progressive decrease in lung impedance variation as airway pressure increased (Figure 4B). This was not the case for the supine-flat position, in which the progressive decrease in impedance variation at higher airway pressures was less pronounced (Figure 4A). This finding further confirms the occurrence of alveolar overdistension at lower airway pressures (i.e., at lower PEEP values) in the semi-recumbent position.

Differences in respiratory and EIT variables observed after 30 min of stabilization are summarized in Table 2. As a consequence of the lower PEEP set in the semi-recumbent position, peak inspiratory, plateau, and mean airway pressure were all significantly lower in patients with 40° head-of-the-bed elevation. Despite the optimization of PEEP, the respiratory system compliance was slightly, but significantly, higher in the supine-flat compared to the semi-recumbent position (48 (38–67) vs. 42 (36–68), mL/cmH_2_O, *p* = 0.005). Similarly, driving pressure was slightly, but significantly, lower in the supine-flat position (9 ± 3 vs. 10 ± 4 cmH_2_O, *p* = 0.005). These differences are likely explained by a higher chest wall compliance in the supine-flat position (*p* = 0.002), as lung compliance was similar between the two angles of trunk inclination (*p* = 0.19).

Despite a lower PEEP after optimization, oxygenation improved in the semi-recumbent position, as demonstrated by a lower venous admixture (34 ± 12 vs. 28 ± 12%, *p* = 0.02), a higher PaO_2_:FiO_2_ (141 ± 46 vs. 196 ± 99, *p* = 0.02) and a lower oxygenation index (14 ± 6 vs. 8 ± 4, *p* < 0.001). CO_2_ clearance was not significantly affected by trunk inclination once PEEP was optimized.

Ventilation distribution assessed with EIT did not differ markedly according to trunk inclination. Nevertheless, the global inhomogeneity index was significantly higher in the supine-flat position (53 ± 11 vs. 46 ± 10, *p* = 0.008), suggesting a more homogenous ventilation distribution in the semi-recumbent position. The difference between end-inspiratory transpulmonary pressure assessed with absolute values and end-inspiratory transpulmonary pressure calculated with the elastance ratio was smaller in the semi-recumbent position (11 ± 3 vs. 4 ± 4 cmH_2_O, *p* < 0.001). This difference is considered a reasonable proxy of the pleural pressure gradient [27]. The end-expiratory absolute transpulmonary pressure was 0 ± 3 cmH_2_O in the supine-flat position and 2 ± 3 cmH_2_O in semi-recumbent position (*p* = 0.06). A significantly different change in EELV (EELI-derived) was observed during the 30-min observation period between the two positions, with EELV being reduced in the supine-flat position and slightly increased in semi-recumbent position. Of note, 60% of lung aeration loss in the supine-flat position occurred in the dorsal, dependent part of the lung.

Central venous pressure was significantly lower in the semi-recumbent position following PEEP optimization (10 ± 3 vs. 4 ± 3 mmH_2_O, *p* < 0.001). No other significant differences in hemodynamics were observed (e-Table S1 of the OSM, Supplementary Materials).

## 4. Discussion

The major finding of our study is that trunk inclination has a marked effect on the respiratory system and that significantly lower PEEP values are required in the semi-recumbent position to optimize respiratory mechanics in mechanically ventilated patients with C-ARDS.

**Figure 5:**
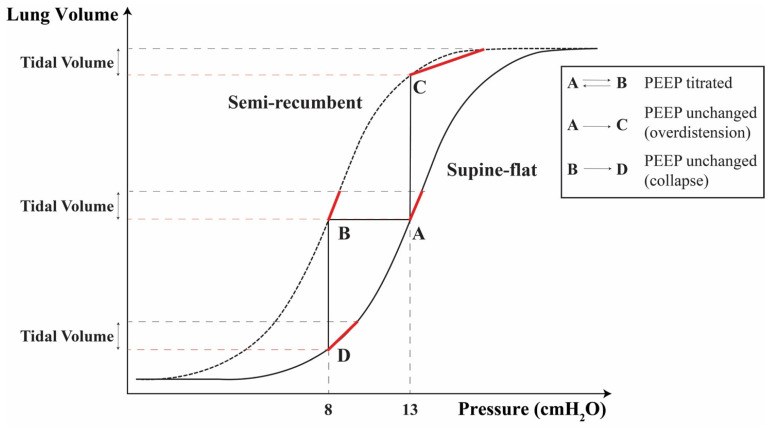
Representation of the implications of a position-related shift of the pressure–volume curve on respiratory mechanics. The theoretical effect on the respiratory system’s pressure–volume curve of the change in trunk inclination in patients on volume-controlled ventilation is represented. On the *Y*-Axis tidal volume is represented as the distance between two consecutive horizontal dashed lines, which represent end-expiratory (red) and end-inspiratory lung volume (black). Please note that the distance between two consecutive horizontal dashed lines is constant along the *Y*-axis, since tidal volume is constant in volume-controlled ventilation. Changing patients’ position from supine-flat (continuous line) to semi-recumbent (short-dashed line), will shift the pressure–volume curve leftwards (and vice versa). If PEEP titration is performed in supine-flat position, a specific “best” PEEP value is identified (in the example 13 cmH_2_O, Point A). If the patient’s trunk inclination is now changed to semi-recumbent, the pressure–volume curve is shifted to the left and, for the same pressure, a higher end-expiratory lung volume (EELV) will be achieved (from A to C). Overdistension might occur, leading to a reduced respiratory system compliance (lower slope of the red-marked part of the pressure-volume curve). On the contrary, if PEEP titration is performed in semi-recumbent position, the identified “best” PEEP will have a lower value (in the example 8 cmH_2_O, Point B). If the patient’s trunk inclination is now changed to supine-flat (without changing PEEP), the pressure-volume curve is shifted to the right and the patient will have a lower EELV for the same pressure (from B to D). Alveolar collapse might occur, potentially leading to a reduced respiratory system compliance. Finally, if trunk inclination is changed from supine-flat to semi-recumbent position and PEEP is optimized (from A to B), a similar EELV will be maintained, resulting in similar compliances of the respiratory system.

One of the main explanations for these findings is the increase in EELV in the semi-recumbent position, shifting the pressure–volume curve of the respiratory system to the left, i.e., having for the same airway pressure a higher gas volume. We did not measure EELV; however, multiple studies have demonstrated the increase in EELV in ARDS patients once placed semi-recumbent [8,9,10]. Furthermore, this hypothesis is upheld by the fact that, during the PEEP trial, alveolar overdistension was present at lower airway pressures in the semi-recumbent compared to the supine-flat position, suggesting that open alveoli had a higher baseline gas volume. Similarly, alveolar collapse started at lower airway pressures compared to the same patients placed supine-flat. This last result is likely attributable to the higher dorsal intra-abdominal pressure that is transmitted to the thoracic cavity in the supine-flat position. The pressure–lung impedance variation curve, a proxy of the pressure-volume curve [28], further corroborates this hypothesis. Indeed, lung impedance increased approximately linearly in the supine-flat position for the explored pressure range. Conversely, clear signs of overdistension were present in semi-recumbent position, with minimal increases in lung impedance despite the reached higher airway pressures, suggesting that total lung capacity was almost reached. Figure 5 schematizes the implications on respiratory mechanics of a position-related shift of the pressure–volume curve.

EIT allowed identifying the PEEP value with the best compromise between alveolar collapse and overdistension [14,15]. Besides having different values according to the employed trunk inclination, the corresponding fractions of alveolar collapse and overdistension were significantly lower in the semi-recumbent position. In other words, in the same patients, the “best” PEEP in the semi-recumbent position was associated with a lower percentage of overdistended and collapsed lung parenchyma. This finding might be explained by a greater tendency towards alveolar collapse in the supine-flat position, due, once again, to the lower starting EELV and to the possible role of the pressure exerted by the abdominal content on the dorsal, dependent lung regions [12,29,30,31,32,33,34,35].

Moreover, we recorded a lower global inhomogeneity index and a smaller pleural pressure gradient [27] in the semi-recumbent position. Taken together, these data point toward a more homogenous ventilation distribution in this position.

As a consequence of the lower PEEP value, mean and plateau airway pressure were significantly lower, with possible implications regarding hemodynamics. Indeed, a lower central venous pressure may improve venous return. Despite the lower PEEP, not only did we observe a significant improvement in oxygenation, as indicated by venous admixture, PaO_2_:FiO_2,_ and the oxygenation index, but we also recorded a lower tendency towards alveolar derecruitment over the 30 min observation period. This assertion is based on the fact that a significant reduction in EELV (EELI-derived) over time was observed in the supine-flat position (mainly dorsal), while it did not change or even slightly increased when patients were placed semi-recumbent. A possible role of end-expiratory transpulmonary pressure is conceivable, as we observed a trend toward higher values in the semi-recumbent position.

Finally, we observed a similar CO_2_ clearance in the two positions. This finding differs from our previous study [11], where CO_2_ clearance worsened in semi-recumbent patients. These data further corroborate the hypothesis of a major role of alveolar overdistension as responsible for worse CO_2_ clearance in semi-recumbent position when PEEP was not optimized.

### 4.1. Clinical Implications

There are several approaches to set PEEP at the bedside [14,36,37], and currently, no technique has been shown to be superior. In some time-consuming methods (e.g., compliance or EIT-based ones), the patient’s position certainly contributes to defining the “best” PEEP. On the contrary, the simpler and frequently applied PEEP-FiO_2_ tables do not consider this aspect [4]. The results of our study support the use of physiology-based PEEP titrations, as these approaches take into account trunk inclination. In addition, our study conveys a straightforward clinical message: physiology-based PEEP titration should be performed in the position in which we plan to ventilate our patient. Indeed, as we demonstrated, performing the PEEP titration in the supine-flat position and applying the identified “best” PEEP in semi-recumbent position will lead to significant overdistension. On the contrary, alveolar collapse could be favored if PEEP was optimized in the semi-recumbent position and the supine-flat position was chosen for long-term ventilation (Figure 5). Of course, patients’ trunk inclination is changed several times during the day, e.g., for nursing and medical procedures. As it is not feasible to perform a PEEP titration at every trunk inclination change, it seems reasonable to propose a bedside “rule of thumb” for PEEP correction: increase PEEP by approximately 1 cmH_2_O for every 10° angle reduction (and vice versa). It is worth mentioning that a certain variability in response to trunk inclination exists and could be explained by different mechanical characteristics of the respiratory system [38].

Similarly to clinical practice, we find it of paramount importance in clinical trials to report the used trunk inclination both for PEEP titration performance and long-term mechanical ventilation [39]. Indeed, if PEEP titration is performed in supine-flat patients and the semi-recumbent position is chosen for mechanical ventilation, this could lead to significant overdistension, potentially increasing the incidence of barotrauma and VILI [40]. In addition to this consideration, it might be of interest to speculate on which trunk inclination could be associated with a lower risk of VILI, provided PEEP is optimized. Our short-term data certainly do not allow us to draw conclusions. However, a lower airway pressure, a lower percentage of overdistention and collapse, more homogenous ventilation, and better oxygenation were recorded in the semi-recumbent position, once PEEP was optimized. In addition, despite some controversies [41], the semi-recumbent position is suggested to reduce the incidence of ventilator-associated pneumonia [42]. Taking these considerations together, we think that there might be some benefit in ventilating patients in the semi-recumbent position, although it is mandatory to optimize PEEP.

### 4.2. Limitations

The fact that only patients with C-ARDS were enrolled is both a strength and a limitation. On the one hand, the population was very homogenous, characterized by respiratory failure with the same etiology. On the other, we might wonder if our findings can be transferred to patients with “classical” ARDS. Despite some possible differences with C-ARDS [43,44] we think that, based on the available literature [8,9] and the current physiological understanding, it is safe to hypothesize a similar behavior in patients with “classical” ARDS. The short-term nature of the study is an additional limitation.

## 5. Conclusions

There is no single “best” PEEP for a given patient, at a given moment. Indeed, the angle of trunk inclination has a major effect on the respiratory system characteristics of mechanically ventilated patients with C-ARDS, with clear clinical implications. In particular, the “best” PEEP is markedly lower in the semi-recumbent than in the supine-flat position. In addition, several aspects, including more homogenous ventilation, better oxygenation, and less derecruitment, suggest that the semi-recumbent position (with optimized PEEP) might have some advantages over the supine-flat position.

## Figures and Tables

**Table 1:** Patients’ characteristics.

Variables	*n* = 12
Age, years	65 ± 8
Male sex, *n* (%)	10 (83)
BMI, Kg/m^2^	28.1 ± 5.1
PBW, Kg	68 ± 8
SAPS II	36 ± 8
Days from onset of symptoms, *n*	12 ± 5
Days from intubation, *n*	3 ± 2
PaO_2_:FiO_2_	155 ± 41
ARDS severity, *n* (%)	
Mild	2 (17)
Moderate	10 (83)
Severe	0 (0)
C_RS_, mL/cmH_2_O	44 ± 15
V_T_/PBW, mL/kg	6.0 ± 0.6
Respiratory rate, *n*/min	21 ± 3
PEEP, cmH_2_O	12 ± 3
FiO_2_	0.67 ± 0.15

BMI = body mass index; PBW = predicted body weight; SAPS II = simplified acute physiology score II; C_RS_ = compliance of the respiratory system; PaO_2_ = partial pressure of oxygen in the arterial blood; PEEP = positive end-expiratory pressure; FiO_2_ = fraction of inspired oxygen. Reported ventilatory settings refer to the clinical settings on the study day. ARDS severity has been evaluated by using the “Berlin” definition for ARDS [16].

**Table 2:** Differences between supine-flat and semi-recumbent position after PEEP titration with EIT.

Variables Measured after 30 min at “Best” PEEP_EIT_	Supine-Flat (0°)	Semi-Recumbent (40°)	*p*
Peak Inspiratory Pressure, cmH_2_O	29 ± 2	25 ± 3	<0.001
Plateau Pressure, cmH_2_O	22 ± 2	19 ± 3	<0.001
Mean Airway Pressure, cmH_2_O	17 ± 1	12 ± 1	<0.001
End–expiratory airway pressure, cmH_2_O	14 ± 2	9 ± 2	<0.001
Driving pressure, cmH_2_O	9 ± 3	10 ± 4	0.005
End–inspiratory esophageal pressure, cmH_2_O	16 ± 2	10 ± 4	<0.001
End–expiratory esophageal pressure, cmH_2_O	14 ± 2	7 ± 4	<0.001
P_L_es, cmH_2_O	7 ± 3	9 ± 5	0.07
P_L_er, cmH_2_O	17 ± 3	13 ± 4	<0.001
Pleural pressure gradient, cmH_2_O	11 ± 3	4 ± 4	<0.001
Driving transpulmonary pressure, cmH_2_O	7 ± 3	7 ± 4	0.64
End-expiratory transpulmonary pressure, cmH_2_O	0 ± 3	2 ± 3	0.06
C_RS_, mL/cmH_2_O	48 (38–67)	42 (36–68)	0.005
C_CW_, mL/cmH_2_O	253 (166–427)	159 (117–197)	0.002
C_LUNG_, mL/cmH_2_O	61 (50–88)	64 (45–116)	0.19
PaO_2_:FiO_2_	141 ± 46	196 ± 99	0.02
Venous admixture, %	34 ± 12	28 ± 12	0.02
Oxygenation index	14 ± 6	8 ± 4	<0.001
PaCO_2_, mmHg	45 ± 4	47 ± 5	0.19
Ventilatory ratio	1.51 ± 0.27	1.56 ± 0.29	0.19
Alveolar dead space, %	30 ± 9	28 ± 11	0.47
Ventral regional ventilation, %	62 ± 8	60 ± 8	0.35
Dorsal regional ventilation, %	38 ± 9	40 ± 8	0.39
Ventral regional compliance, mL/cmH_2_O	25.9 (22.8–38.7)	23.3 (16.8–36.5)	0.03
Dorsal regional compliance, mL/cmH_2_O	18.1 (9.9–30)	18.9 (8.4–22.4)	0.49
Global Inhomogeneity index	53 ± 11	46 ± 10	0.008
ΔEELV, mL	−153 ± 162	27 ± 203	0.007
Ventral region, mL	−61 ± 94	−30 ± 127	0.39
Dorsal region, mL	−91 ± 80	57 ± 89	<0.001

P_Les_  =  end-inspiratory transpulmonary pressure calculated from esophageal pressure [27]; P_Ler_  =  end-inspiratory transpulmonary pressure calculated from elastance ratio [27]; pleural pressure gradient = PLer − Ples [27]; C_RS_ = respiratory system compliance; C_CW_ = chest wall compliance; C_LUNG_ = lung compliance; FiO_2_ = fraction of inspired oxygen; PaO_2_ = partial pressure of oxygen in the arterial blood; PaCO_2_ = partial pressure of carbon dioxide in the arterial blood; EIT = electrical impedance tomography; ∆EELV = change in end-expiratory lung volume (30 min–0 min). EIT data refer to 11 patients, as in 1 patient; for technical reasons, EIT parameters were not recorded.

## Data Availability

The data that support the findings of this study are available from the corresponding author, [T.L.], upon reasonable request.

## Supplementary Materials

The following supporting information can be downloaded at: https://www.mdpi.com/article/10.3390/jcm12123914/s1, e-Table S1: Difference of hemodynamics and blood gas analysis between supine-flat and semi-recumbent position after PEEP titration. References [45,46] are cited in the Supplementary Materials.
