# Supplementary material for: PEEP Titration Is Markedly Affected by Trunk Inclination in Mechanically Ventilated Patients with COVID-19 ARDS: A Physiologic, Cross-Over Study
Source: J Clin Med. 2023 Jun 8;12(12):3914. doi: 10.3390/jcm12123914 (PMC10299565; doi:10.3390/jcm12123914)
Supplement: Supplementary file 1 [file jcm-12-03914-s001.zip › jcm-2387364-supplementary.pdf]

PEEP titration is markedly affected by trunk inclination in mechanically ventilated patients with COVID-19 ARDS: a physiologic, cross-over study.

Francesco Marrazzo, MD*[1] (ORCID: 0000-0001-5407-6615); Stefano Spina, MD*[1] (ORCID: 0000-0003-3210-1714); Francesco Zadek, MD[2] (ORCID: 0000-0002-2377-3992); Clarissa Forlini, MD[1] (ORCID: 0000-0002-1858-1080); Gabriele Bassi, MD[1]; Riccardo Giudici, MD[1] (ORCID: 0000-0002-2202-6194); Giacomo Bellani, MD[2,3] (ORCID: 0000-0002-3089-205X); Roberto Fumagalli, MD[1,2] (ORCID: 0000-0003-0398-1329); Thomas Langer, MD[1,2] (ORCID: 0000-0002-9725-8520)

* These authors share first authorship

Name of Department(s) and Institution(s):

1. Department of Anesthesia and Critical Care, ASST Grande Ospedale Metropolitano Niguarda, Milano, Italy

2. School of Medicine and Surgery, University of Milano-Bicocca, Milano, Italy

3. APSS Trento, Santa Chiara Hospital, Department of Anesthesia and Intensive Care 1, Trento, Italy

Online Supplementary Material

Calculation of physiologic variables 3

- Ventilatory parameters 3
- Gas exchange 5
- Electrical Impedance Tomography (EIT) 7

Additional results 9

Additional References 10

Calculation of physiologic variables

Ventilatory parameters

Driving pressure was calculated as the difference between plateau airway pressure (Pplat) and total end-expiratory pressure ($PEEP_{TOT}$), both measured during a respiratory hold (inspiratory and expiratory, respectively)

$$Driving\ pressure = Pplat - PEEP_{TOT} \quad \text{(Eq. 1)}$$

Driving pressure throughout the decremental peep trial (**figure 1, Panel C and D**), was calculated by using the plateau pressure during a brief inspiratory pause added at the end of inspiration in volume-controlled ventilation.

Static end-expiratory trans-pulmonary pressure (PTP_{PEEP}) was calculated from total end-expiratory airway pressure ($PEEP_{TOT}$) and end-expiratory esophageal pressure (PES_{PEEP})

$$PTP_{PEEP} = PEEP_{TOT} - PES_{PEEP} \quad \text{(Eq. 2)}$$

Static end-inspiratory transpulmonary pressure (PTP_{Plat}) was computed in two ways:

(I) First, as the absolute difference between Pplat and end-inspiratory esophageal pressure (PES_{Plat}), labeled as $PTP_{Plat, Es}$

$$PTP_{Plat, Es} = Pplat - PES_{Plat} \quad \text{(Eq. 3)}$$

(II) Second, by using the elastance ratio method ($PTP_{Plat, ER}$), which consists of multiplying Pplat by the ratio between lung elastance (E_{LUNG}) and respiratory system elastance (E_{RS})

$$PTP_{Plat, ER} = Pplat \times (E_{LUNG} / E_{RS}) \quad \text{(Eq. 4)}$$

The difference between end-inspiratory transpulmonary pressure assessed with the elastance ratio method ($PTP_{Plat, ER}$) and the absolute esophageal pressure value ($PTP_{Plat, Es}$) was calculated as a proxy of the pleural pressure gradient[1]:

$$Pleural\ pressure\ gradient = PTP_{Plat, ER} - PTP_{Plat, Es} \quad \text{(Eq. 5)}$$

Driving Transpulmonary pressure was calculated as the difference between end-inspiratory transpulmonary pressure ($PTP_{Plat, Es}$) minus end-expiratory trans-pulmonary pressure (PTP_{PEEP})

$$Driving\ transpulmonary\ pressure = PTP_{Plat, Es} - PTP_{PEEP} \quad \text{(Eq. 6)}$$

Compliance of the respiratory system (C_{RS}), of the chest wall (C_{CW}), and of the lung (C_{LUNG}) were calculated as follows:

$$C_{RS} = Tidal\ volume\ (ml) / Driving\ pressure\ (cmH_2O) \quad \text{(Eq. 7)}$$

$$C_{CW} = Tidal\ volume\ (ml) / \Delta PES\ (cmH_2O) \quad \text{(Eq. 8)}$$

$$C_{LUNG} = Tidal\ volume\ (ml) / Driving\ transpulmonary\ pressure\ (cmH_2O) \quad \text{(Eq. 9)}$$

<u>Gas exchange</u>

Venous admixture, (%) (Qs/Qt) was calculated as:

$$\frac{\dot{Q}s}{\dot{Q}t} = \frac{CcO_2 - CaO_2}{CcO_2 - CvO_2} \qquad \text{(Eq. 10)}$$

Where CaO_2 and CvO_2 are the contents of oxygen for arterial and central venous blood and CcO_2 is the oxygen content of capillary blood, calculated as:

$$CcO_2 = [Hb] \times 1.39 \times 1 + [PAO_2 \times 0.003]$$

Where PAO_2 is the alveolar partial pressure of oxygen expressed in mmHg and calculated as:

$$PAO_2 = FiO_2 \times (Patm - 47) - \frac{PaCO_2}{RQ} \qquad \text{(Eq. 11)}$$

Where Patm is the atmospheric pressure, 47 is water vapor pressure, $PaCO_2$ is the partial pressure of CO_2 in arterial blood and RQ is the respiratory quotient, assumed as 0.84.

Alveolar dead space (V_D/V_T) was calculated as:

$$\text{Alveolar dead space, \%} = [(PaCO_2 - PEtCO_2) / PaCO_2] * 100 \qquad \text{(Eq. 12)}$$

Ventilatory ratio (VR) was calculated as follows[2]:

$$VR = (Minute\ ventilation \times PaCO_2)/(predicted\ body\ weight \times 100 \times 37.5) \quad \text{(Eq. 13)}$$

where minute ventilation is expressed in ml/min and $PaCO_2$ in mmHg.

The Oxygenation Index (OI) was calculated as follows[3]:

$$OI = (Mean\ airway\ pressure\ x\ FiO_2)\ /\ PaO_2 \quad \text{(Eq. 14)}$$

Where mean airway pressure is expressed in cmH_2O, FiO_2 is expressed as a percentage and PaO_2 is the arterial partial pressure of oxygen, expressed in mmHg.

<u>Electrical Impedance Tomography (EIT)</u>

Regional compliance based on EIT data was measured as follows:

$Ventral\ C_{RS} = Ventral\ Tidal\ Volume\ /\ Driving\ pressure$ (Eq. 15)

$Dorsal\ C_{RS} = Dorsal\ Tidal\ Volume\ /\ Driving\ pressure$ (Eq. 16)

Regional tidal volume is derived from EIT images, multiplying tidal volume by the percentage of ventilation of the ventral/dorsal region of interest. Both compliances are expressed in ml/cmH_2O.

In the PEEP-titrated part, the difference in End-Expiratory Lung Volume (EELV) between 30 minutes and 0 minutes was measured by using End-Expiratory Lung Impedance (EELI, Impedance units) as follows[4,5]:

$Global\ \Delta EELV_{30\text{-}0\ min} = (EELI_{30\ min} – EELI_{0\ min})\ x\ (VT\ /VT_{EIT})$ (Eq. 17)

Where EELV is expressed in ml, EELI refers to the global end-expiratory lung impedance (expressed in arbitrary units) measured at 30 minutes and at 0 minutes, VT_{EIT} refers to the tidal impedance change (expressed in arbitrary units) and VT refers to the tidal volume delivered by the ventilator, expressed in ml.

$Ventral\ \Delta EELV_{30\text{-}0\ min} = (EELI_{30\ min} – EELI_{0\ min})\ x\ (VT\ /VT_{EIT})$ (Eq. 18)

Where EELV is expressed in ml, EELI refers to the ventral end-expiratory lung impedance (expressed in arbitrary units) measured at 30 minutes and at 0 minutes, VT_{EIT} refers to the tidal

impedance change (expressed in arbitrary units), and VT refers to the tidal volume delivered by the ventilator, expressed in ml.

$$Dorsal\ \Delta EELV_{30\text{-}0\ min} = (EELI_{30\ min} - EELI_{0\ min})\ x\ (VT / VT_{EIT}) \qquad \text{(Eq. 19)}$$

Where EELV is expressed in ml, EELI refers to the dorsal end-expiratory lung impedance (expressed in arbitrary units) measured at 30 minutes and at 0 minutes, VT_{EIT} refers to the tidal impedance change (expressed in arbitrary units), and VT refers to the tidal volume delivered by the ventilator, expressed in ml.

Additional results

e-Table S1. Difference of hemodynamics and blood gas analysis between supine-flat and semi-recumbent position after PEEP titration.

	Supine–flat (0°)	Semi–recumbent (40°)	*P*
**Hemodynamics and blood gas analysis**			
SvO_2, %	76±6	76±6	0.76
pH	7.39±0.05	7.38±0.05	0.03
Lactate, mmol/L	1.2±0.3	1.2±0.3	0.52
Difference $PvCO_2$ - $PaCO_2$, mmHg	7±4	7±5	0.47
HR, n/min	76±20	75±20	0.77
MAP, mmHg	82±12	78±12	0.20
CVP, mmHg	10±3	4±3	<0.001

Data are expressed as mean±SD and refer to 12 patients. SvO_2 = venous oxygen saturation; $PaCO_2$ = partial pressure of carbon dioxide in the arterial blood; $PvCO_2$ = partial pressure of carbon dioxide in the venous blood; HR = heart rate; MAP = mean arterial pressure; CVP = central venous pressure;

Additional References

1. Yoshida, T.; Amato, M.B.P.; Grieco, D.L.; Chen, L.; Lima, C.A.S.; Roldan, R.; Morais, C.C.A.; Gomes, S.; Costa, E.L.V.; Cardoso, P.F.G.; et al. Esophageal Manometry and Regional Transpulmonary Pressure in Lung Injury. *Am J Respir Crit Care Med* 2018, *197*, 1018–1026, doi:10.1164/rccm.201709-1806OC.
2. Sinha, P.; Calfee, C.S.; Beitler, J.R.; Soni, N.; Ho, K.; Matthay, M.A.; Kallet, R.H. Physiologic Analysis and Clinical Performance of the Ventilatory Ratio in Acute Respiratory Distress Syndrome. *Am J Respir Crit Care Med* 2019, *199*, 333–341, doi:10.1164/rccm.201804-0692OC.
3. Hussain, T.; Braithwaite, I.; Hancock, S. Errors and Inaccuracies in Internet Medical Calculator Applications: An Example Using Oxygenation Index. *Archives of Disease in Childhood* 2018, doi:10.1136/archdischild-2018-315323.
4. Hinz, J.; Hahn, G.; Neumann, P.; Sydow, M.; Mohrenweiser, P.; Hellige, G.; Burchardi, H. End-Expiratory Lung Impedance Change Enables Bedside Monitoring of End-Expiratory Lung Volume Change. *Intensive Care Med* 2003, *29*, 37–43, doi:10.1007/s00134-002-1555-4.
5. Mauri, T.; Eronia, N.; Turrini, C.; Battistini, M.; Grasselli, G.; Rona, R.; Volta, C.A.; Bellani, G.; Pesenti, A. Bedside Assessment of the Effects of Positive End-Expiratory Pressure on Lung Inflation and Recruitment by the Helium Dilution Technique and Electrical Impedance Tomography. *Intensive Care Med* 2016, *42*, 1576–1587, doi:10.1007/s00134-016-4467-4.
